# Changes of Cytokines during a Spaceflight Analog - a 45-Day Head-Down Bed Rest

**DOI:** 10.1371/journal.pone.0077401

**Published:** 2013-10-15

**Authors:** Xi Xu, Cheng Tan, Pingping Li, Shusong Zhang, Xuewen Pang, Hongju Liu, Li Li, Xiuyuan Sun, Yu Zhang, Hounan Wu, Xiaoping Chen, Qing Ge

**Affiliations:** 1 Key Laboratory of Medical Immunology, Ministry of Health, Department of Immunology, School of Basic Medical Sciences, Peking University Health Sciences Center, Beijing, P. R. China; 2 State Key Laboratory of Space Medicine Fundamentals and Application, Chinese Astronaut Research and Training Center, Beijing, P. R. China; 3 Peking University Medical and Health Analytical Center, Peking University Health Science Center, Beijing, P. R. China; Glaxo Smith Kline, Denmark

## Abstract

Spaceflight is associated with deregulation in the immune system. Head-down bed rest (HDBR) at -6° is believed to be the most practical model for examining multi-system responses to microgravity in humans during spaceflight. In the present study, a 45-day HDBR was performed to investigate the alterations in human immune cell distributions and their functions in response to various stimuli. The effect of countermeasure, *Rhodiola rosea* (RR) treatment, was also examined. A significant decrease of interferon-γ (IFN-γ) and interleukin-17 (IL-17) productions by activated T cells, increase of IL-1β and IL-18 by activated B and myeloid cells were observed during HDBR. The upregulation of serum cortisol was correlated with the changes of IL-1 family cytokines. In addition, a significant increase of memory T and B cell and regulatory T cells (Treg) were also detected. The uptake of RR further decreased IFN-γ level and slowed down the upregulation of IL-1 family cytokines. These data suggest that for prolonged HDBR and spaceflight, the decreased protective T cell immunity and enhanced proinflammatory cytokines should be closely monitored. The treatment with RR may play an important role in suppressing proinflammatory cytokines but not in boosting protective T cell immunity.

## Introduction

The changes of immune system during short- or long-duration of spaceflights include altered leukocyte distribution, altered serum cytokine level, reduced functions of natural killer (NK) cell, granulocyte, and monocyte, reduced leukocyte proliferation following activation, decreased delayed-type hypersensitivity to recall antigens, and latent viral reactivation [1-24]. Physiological and psychological stresses, microgravity, vibration exposure, disrupted circadian rhythms, impaired nutrition, and radiation were thought to contribute to the deregulation in immunity [24]. Although the existence of clinical risks related to such flight-associated dysfunction of immune surveillance has not been firmly concluded, concerns of the occurrence of malignant and autoimmune diseases in long-duration spaceflights are growing. Strategies of monitoring the immune system and countermeasures have been a great interest to many investigators. 

Due to the difficulties in performing in-flight human physiology research, several ground-based spaceflight analogs have been developed. Among them, the head-down bed rest (HDBR) of -6° is determined to be the best by NASA, representing the most practical model for examining multi-system responses to microgravity in humans during spaceflight [14]. Using this model, various immnue alterations have been reported. Some of them mimic the changes found in astronauts [14,25-30], such as a gradual decrease in the number of IFN-γ-producing T cells and Cytomegalovirus- and Epstein-Barr virus-specific T cells. However, many such studies focused on the percentages of immune cell populations, cytokines in the serum, proliferation and cytotoxicities of T and NK cells. The production of inflammatory cytokine milieu by immune cells upon various stimuli, the subpopulations of γδ T cells and B cells have seldom been examined. In addition, the impact of adaptogen-based countermeasures on immunity under microgravity has not been tested. 

*Rhodiola rosea* (RR), a type of adaptogen, has been used as traditional medicine in parts of Europe, Asia, and Russia for centuries [31]. Although the active constituents in RR are currently unclear, the common indications include performance enhancement, fatigue reduction, and alleviation of depression symptoms [31]. An immunostimulating potential was also found in rodents *in vivo* and human peripheral mononuclear cells (PBMCs) *in vitro*. RR, or a major component of RR, salidroside, could promote lymphocyte proliferation, cytokine and antibody productions [32-36]. 

To investigate the immune changes during and after a long-term spaceflight simulation and to examine whether RR has an impact on the immune system, a placebo- and an RR-treated groups were set up for a 45-day HDBR. The changes in peripheral mononuclear cells, in particular, the cytokine production function of various immune cells, were monitored. 

## Results

### Changes of cytokine production patterns of PBMCs upon various stimuli

**Figure 1:**
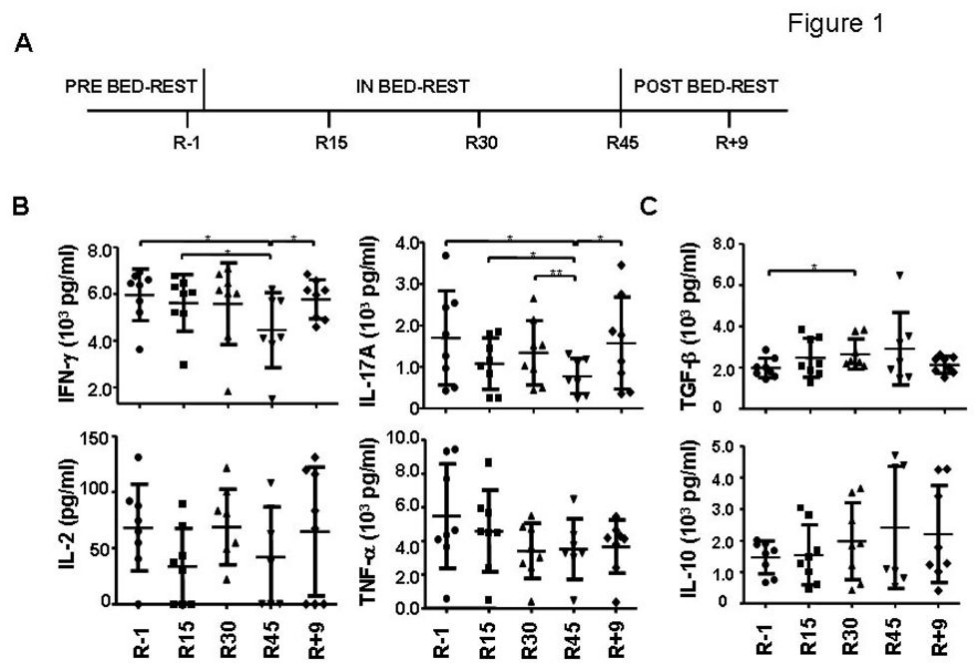
Changes of T cell-derived cytokines during HDBR. (A) Scheme of the 45-day HDBR of -6° and the time of sample collections. (B) Changes of T cell-derived cytokines. PBMCs were stimulated by anti-CD3 and anti-CD28 antibodies for 2 days. The supernatants were analyzed by cytometric bead array for cytokines IFN-γ, IL-17A, IL-2, TNF-α (B) and TGF-β1, IL-10 (C). The average level of each cytokine at each time point was shown. The statistical significance between any two time points within the control group was calculated by two-tailed paired Student *t* test. The following terminology is used to denote *p* values calculated by Student *t* test: *p<0.05, **p<0.01, ***p<0.005.

We focused on the cytokine milieu produced by various immune cells. Cytokines produced by activated T cells were examined after stimulation of PBMCs with anti-CD3 and anti-CD28 for 2 days. The levels of T cell-derived effector cytokines such as IFN-γ and IL-17A showed a gradual decrease during the HDBR, reaching the lowest level on R45 (Figure 1B, *p* = 0.05, 0.003, respectively by repeated measures ANOVA, the statistical significance of the data between time points was shown in the figure; 25.0%±26.2% and 53.8%±20.3% decreased on R45 as compared to R-1, respectively). Unlike the findings in post-flight and a recent HDBR studies, we did not find a significant decrease in IL-2 expression (Figure 1B) [2,22,23,37]. No consistent and significant changes were found in the production of IL-4, IL-22, TNF-α, and IL-6 by T cells (Figure 1B, Figure S1A, and data not shown).

The T cell-derived cytokines with regulatory roles, such as TGF-β1 and IL-10 showed a slight upregulation during HDBR, reaching 47% (TGF-β1) and 62% (IL-10) increase when the cytokine levels from R-1 and R45 were compared (Figure 1 and data not shown). The statistical analysis of these two cytokines, however, did not reach *p*<0.05, probably due to the variations within the data and the small number of subjects. 

**Figure 2:**
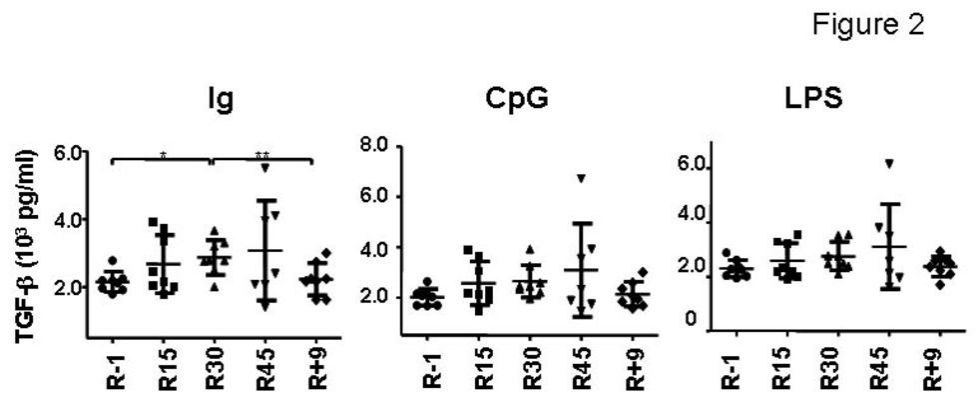
Production of TGF-β1 by immune cells. PBMCs were stimulated by antibodies against immunoglobulin or CpG for 3 days, or LPS for 2 days. The supernatants were examined for TGF-β1 concentration.

Regulatory cytokines produced by other immune cells were also tested. B cell-derived ones were examined by anti-human IgA+IgG+IgM (H+L) stimulation of PBMCs for 3 days. A fraction of PBMCs was also treated with 2.5 μg/ml CpG-B or 1 μg/ml lipopolysaccharide (LPS). B cells, monocytes, and dendritic cells can be activated by CpG-B whereas the latter two types of cells can be activated by small dose of LPS [38,39]. Similar to the results from T cells, a trend of gradual increase of TGF-β1 was found by all three stimuli, in particular, by anti-human antibody stimulation (Figure 2). Although no significant differences were found by statistical analysis, the production of this cytokine reached the highest level on R45 and dropped on R+9 to the level comparable to R-1. 

**Figure 3:**
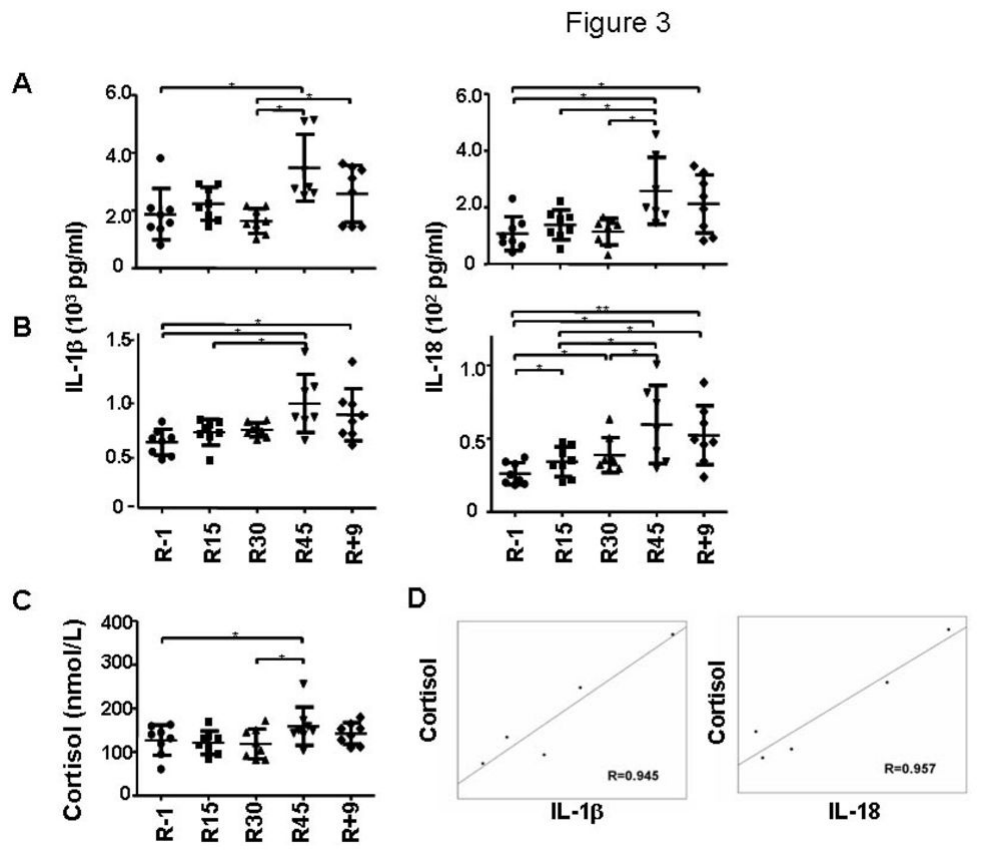
Changes of IL-1β and IL-18 productions and serum cortisol level during HDBR. PBMCs samples were stimulated with CpG for 3 days (A) and LPS for 2 days (B). The supernatants were then collected and the concentrations of IL-1β and IL-18 were measured. The serum cortisol levels during HDBR were measured (C) and its correlations with CpG-stimulated IL-1β and IL-18 production were determined (D).

The production of proinflammatory cytokines by PBMCs upon CpG-B or LPS stimulations was also examined. Interestingly, the levels of IL-1β and IL-18 produced by CpG- or LPS-stimulated PBMCs were upregulated significantly, especially at the time point of R45 (121.4%±112.3% increase of IL-1β by CpG; 67.5%±56.5% of IL-1β by LPS; 211.0%±26.2% of IL-18 by CpG; 158.5%±123.3% of IL-18 by LPS). Upon completion of bed rest, the production of these cytokines started to decline (Figure 3A-B, analysis by repeated measures ANOVA showed that *p* = 0.003 and 0.004 for IL-1β by CpG and LPS, respectively; *p* = 0.002 and 0.000 for IL-18 by CpG and LPS, respectively; the statistical significance between time points was shown in the figure). A mild decrease of MCP-1 was found in LPS-stimulated PBMCs but no statistical significance was reached (data not shown). Other cytokines such as IL-12, TNF-α, IL-10, IL-8, IL-6, and IL-1Ra did not show consistent and significant changes (Figure S1B (CpG) and Figure S1C (LPS) and data not shown).

The changes of IL-1 production under sterile condition may be affected by the activation of neuronal pathways such as the hypothalamo-pituitary-adrenal axis (HPA) and the sympathetic nervous system (SNS). For instance, the increase of cortisol and catecholamine levels have been observed in astronauts prior to launch, at landing, and sometimes during the spaceflight [13,40-44]. Cortisol can suppress the production of proinflammatory cytokines such as IL-1. It may also sensitize myeloid cells to produce more proinflammatory cytokines upon immunologic challenges [45-47]. On the other hand, IL-1β was found to play an important role in upregulation of the activity of HPA axis [48-51]. Thus, we measured the serum levels of cortisol and investigated its correlation with cytokine productions. As shown in Figure 3C, the concentration of cortisol in the serum was highest on R45, reaching a significance of *p* = 0.029. The correlation between CpG-stimulated IL-1β and IL-18 production was very high, with R = 0.945 (*p* = 0.015) and R = 0.957 (*p* = 0.011), respectively (Figure 3D). A significant correlation was also found between LPS-stimulated IL-1β production and cortisol (R = 0.882, *p* = 0.047, data not shown). No correlation was found between cortisol and other cytokines. 

### Changes in serum cytokines

**Figure 4:**
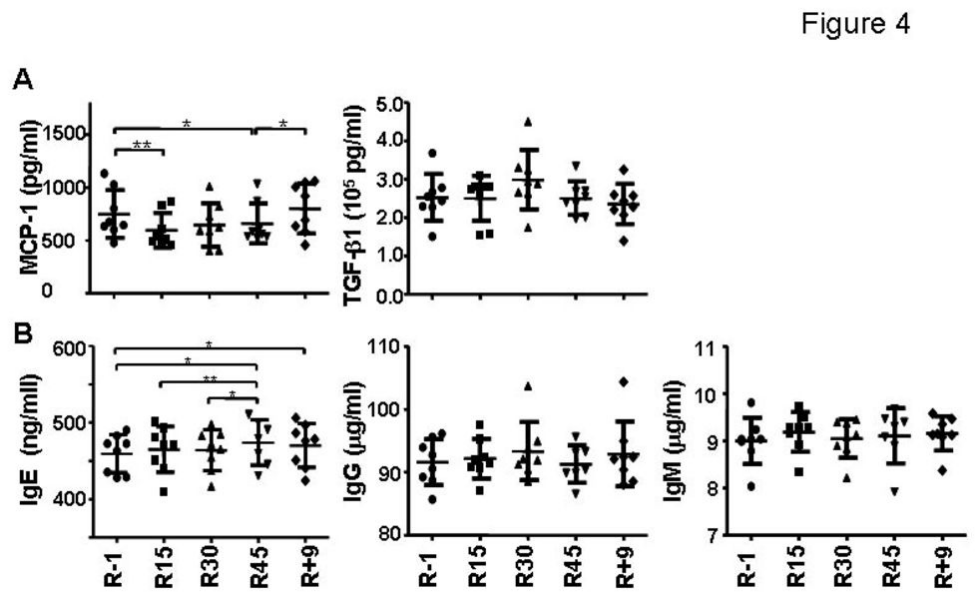
Changes of serum cytokine concentration and immunoglobulin production by CpG-stimulated B cells during HDBR. (A) Serum MCP-1 and TGF-β1 concentration. Serum samples were collected and Searchlight Multiplex immuno assay kits were used to measure the concentration of MCP-1. (B) Analysis of CpG-stimulated IgE, IgG, and IgM production during HDBR.

Multiple cytokines in the serum were examined. The levels of monocyte chemotactic protein-1 (MCP-1, a macrophage chemoattractant) had a quick and significant decrease within 15 days of bed rest and remained low until the completion of HDBR (Figure 4A, *p* = 0.002 by repeated measures ANOVA). The serum concentration of MCP-1 was recovered on R+9. No significant changes were found in IL-10, IL-1β, IL-15, and TGF-β1 (Figure 4A and data not shown). The serum concentration of IFN-γ, IL-4, IL-6, TNF-α, IL-2 were not detectable in some of the volunteers (data not shown).

### Changes of antibody production by B cells upon CpG stimulation

We tested the antibody production function of activated/memory B cells. Supernatants from CpG-stimulated PBMCs were collected and the levels of IgG, IgM, IgA, and IgE were measured by ELISA. Only the level of IgE showed a significant increase on R45 (Figure 4B, *p* = 0.039 by repeated measures ANOVA, the statistical significance between time points was shown in the figure). 

### Changes in cell subsets

**Figure 5:**
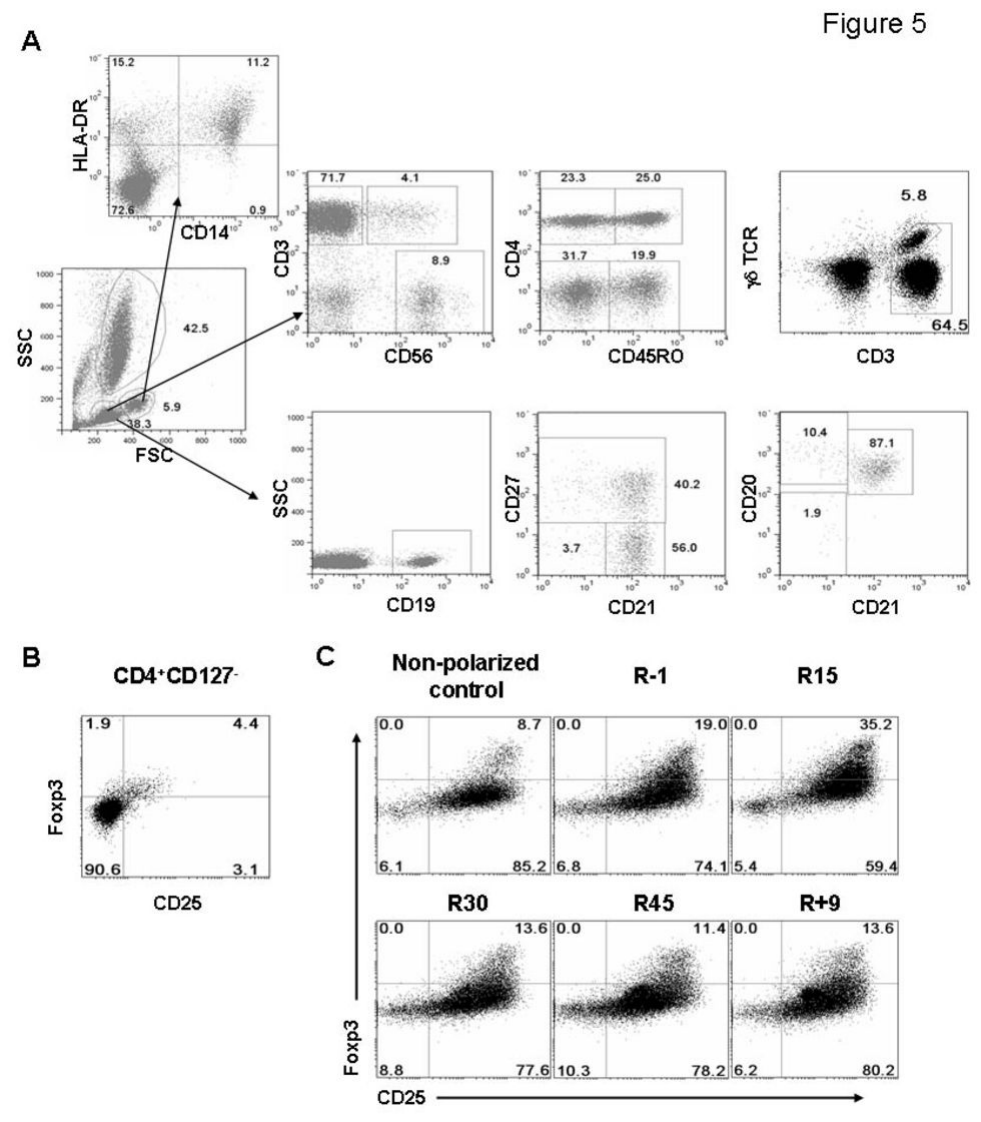
Immune cell subset analysis by flow cytometry. (A) The analysis of immune cell subsets from peripheral blood. Lymphocytes were first gated from forward and side scatter. CD3^+^CD56^-^ cells were then gated as conventional T cells. Within this population, naïve T cells were CD4^+^CD45RO^-^ or CD4-CD45RO^-^ (or termed as CD8^+^ naïve T cells); memory T cells were CD4^+^CD45RO^+^ or CD4^-^CD45RO^+^ (or termed as CD8^+^ memory T cells). NKT, CD3^+^CD56^+^ and NK, CD3^-^CD56^+^. The expression of CD19 was used to analyze B cells. Within CD19^+^ cells, CD21^+^CD27^-^ cells were naïve B cells; CD21^-^CD27^-^ were tissue-like memory B cells; and CD27^+^ were classical memory B cells. The CD27^+^ cells were further divided into CD21^+^CD20^+^ resting memory B, CD21^-^CD20^+^ activated memory B; and CD21^-^CD20^-^ plasmablasts. (B) The analysis of Treg cells in PBMCs. Lymphocytes were stained with CD4, CD127, CD25, and Foxp3. The CD25 and Foxp3 expression of CD4^+^CD127^-^ cells was shown. (C) The analysis of iTreg differentiation in vitro. PBMCs were stimulated with anti-CD3 and anti-CD28 in the presence of TGF-β1 and IL-2 for 5 days. The cells were then stained with antibodies and the percentages of CD4^+^CD25^+^Foxp3^+^ cells were calculated. The plot showed the results of one representative volunteer at 5 different time points. The non-polarized control was stimulated with anti-CD3 and anti-CD28 for 5 days without the addition of polarizing cytokines.

The percentages of total T cells, CD4^+^ and CD8^+^ T cells in the peripheral blood did not change significantly until 9 days after the completion of bed rest (R+9) (Table 1, Figure 5A and Figure S2). These include a significant increase of total T and CD4^+^ T cells and a decrease of CD8^+^ T cells on R+9. Consistently, about 14% increase of CD4-to-CD8 ratio on R+9 was seen as compared with R45 (Table 1 and Figure S2). A mild decrease of γδ T cells and NK cells was found 9 days after HDBR (Table 1, Figure 5A and Figure S2). 

**Table 1:** Statistical analysis of the distribution of various immune cells.

Immune cells	((R15)/(R-1)-1)x 100%	((R45)/(R-1)-1) x 100%	((R+9)/(R45)-1) x 100%	Repeated measures ANOVA (*p* values)
Total T	↓3.7 ± 6.9^a^	↓2.4 ± 4.9	↑6.2 ± 4.9	0.011
CD4^+^ T	↓0.6 ± 3.2	↓2.0 ± 5.9	↑5.5 ± 4.8	0.009
CD8^+^ T	↑1.2 ± 4.2	↑3.1 ± 6.8	↓6.8 ± 5.8	0.009
CD4/CD8 ratio	↓1.6 ± 7.3	↓4.2 ± 11.8	↑13.8 ± 12.0	0.004
NK	↑9.4 ± 42.4	↑38.7 ± 56.5	↓36.5 ± 17.8	0.050
γδ	↑29.1 ± 64.7	↑35.3 ± 33.2	↓23.6 ± 17.1	0.056
CD27^+^ γδ T	↑12.2 ± 76.4	↓5.0 ± 10.2	↑5.3 ± 9.2	0.510
CD94^+^ γδ T	↑50.3 ± 187.2	↓2.5 ± 10.9	↓4.3 ± 13.3	0.157
Naïve CD4^+^ T	↓8.1 ± 7.5	↓11.6 ± 3.9	↑13.2 ± 7.5	0.000
Memory CD4^+^ T	↑5.4 ± 6.3	↑7.1 ± 7.2	↓1.5 ±4.6	0.022
Naïve/memory CD4^+^ ratio	↓15.5 ± 14.3	↓21.4 ± 9.7	↑12.6 ± 6.8	0.000
Naïve CD8^+^ T	↓5.4 ± 5.0	↓5.0 ± 5.8	↑3.3 ± 4.8	0.008
Memory CD8^+^ T	↑11.7 ± 11.9	↑22.2 ± 18.8	↓21.0 ± 10.7	0.010
Naïve/memory CD8^+^ ratio	↓18.6 ± 16.8	↓29.9 ± 27.3	↑23.5 ± 10.5	0.004
Circulating Treg	↓5.8 ± 29.4	↑22.5 ± 15.7	↓28.2 ± 38.5	0.026
iTreg	↑34.0 ± 29.7	↓8.5 ± 19.0	↑6.8 ± 21.0	0.051
Naïve B	↓8.5 ± 8.7	↓8.6 ± 6.9	↓0.5 ± 8.0	0.007
Classical memory B	↑6.6 ± 9.2	↑12.8 ± 8.5	↑1.8 ± 7.4	0.000
Naïve/memory B ratio	↓18.5 ± 23.6	↓24.6 ± 19.1	↓3.4 ± 15.6	0.001
Resting memory B	↑2.6 ± 10.8	↑19.8 ± 9.9	↑1.7 ± 6.7	0.000
Activated memory B	↑25.7 ± 15.3	↓12.1 ± 17.2	↑1.0 ± 21.1	0.000
Tissue like memory B	↑31.6 ± 13.0	↓12.6 ± 10.0	↓16.8 ± 14.4	0.022
CD14^+^ monocyte	↓5.0 ± 19.3	↑30.5 ± 20.5	↓0.9 ± 21.9	0.002
MHC II^+^CD14^+^ monocyte	↓11.6 ± 16.6	↑22.2 ± 18.4	↓3.3 ± 20.3	0.000

a increased (

↑) or decreased (↓) ratio, Mean % ± Standard deviation %

**Figure 6:**
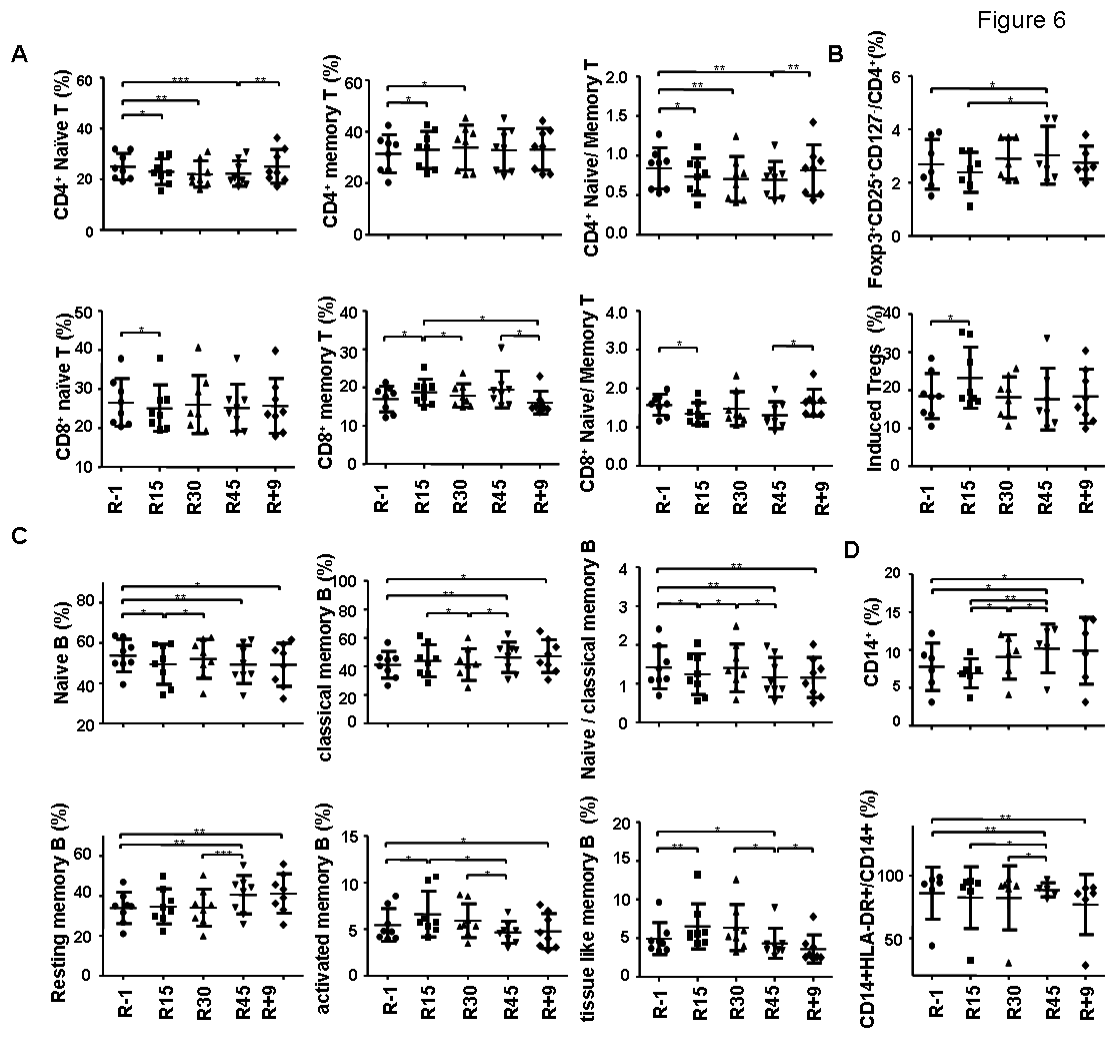
The alteration of various immune cell percentages during HDBR. (A) The analysis of naïve and memory T cell percentages in CD4^+^ and CD8^+^ T cell subsets. (B) The changes of Treg percentage in peripheral T cells and iTreg differentiation *in* *vitro*. (C) The analysis of B cell subsets. (D) The analysis of monocytes. The average percentage of each cell population at each time point was shown. The statistical significance between any two time points within the control group was analyzed by two-tailed paired Student *t* test. The following is used to denote *p* values: *p<0.05, **p<0.01, ***p<0.005.

When T cell subsets were analyzed, a significant decrease of naïve CD4^+^ T cell percentages (CD45RO^-^, 12%±5.8% decreased on R30 as compared R-1) and an increase of memory CD4^+^ T percentages (CD45RO^+^, 7.4%±8.3% increased on R30 as compared to R-1) were clearly found as early as R15 and further changed on R30. The percentage of naïve but not memory CD4^+^ T cells then bounced back on R+9 (Figure 5A, 6A, Table 1). The naïve-to-memory CD4^+^ T cell ratio was thus reduced significantly during the period of HDBR (Figure 6A, Table 1, 22.8%±14.5% decreased on R30 as compared to R-1). A similar increase of memory T and a decrease of naïve-to-memory ratio were observed in CD8^+^ T cells during HDBR (Figure 5A, 6A, Table 1). The changes of regulatory T cells (Treg) were also monitored. An increase of CD4^+^CD25^+^Foxp3^+^CD127^-^ Treg cells in total circulating T cells was found on R45 (Figure 5B, 6B, Table 1). The percentage of Treg cells returned to the baseline on R+9. Compared to the late increase of Tregs in the circulation, an upregulation of induced Treg (iTreg) was seen from the R15 but not R45 samples (Figure 5B, 6B, Table 1). The differentiation of induced Treg (iTreg) at various time points was derived from the stimulation of peripheral blood mononuclear cells (PBMCs) with anti-CD3, anti-CD28, and TGF-β1 for 3 days. 

Similar to the changes in T cells, the percentage of classical memory B showed a significant increase during HDBR and was maintained at a high level even 9 days after completion (Figure 6C, Table 1, 12.8%±8.5% increased on R45 as compared to R-1). As naïve B cell percentage decreased (8.6%±6.9% decreased on R45 as compared to R-1), a significant decrease of naïve-to-memory B cell ratio was thus seen (Figure 6C, Table 1 24.6%±19.1% decreased on R45 as compared to R-1). Among memory B cells, the percentage of CD27^+^CD21^+^ resting memory B cells increased significantly on R45 and remained high on R+9 (Figure 6C, Table 1). Activated (CD27^+^CD21^-^CD20^hi^) and tissue like (CD27^-^CD21^-^) memory B cells, however, increased at the early phase (R15) but gradually decreased during the bed rest, reaching the lowest numbers on R45 (Figure 6C, Table 1). The percentage of activated memory B cells was not recovered on R+9. 

A significant increase of CD14^+^ monocytes was observed during HDBR. The percentage of CD14^+^ monocytes reached the highest on R45 and remained high on R+9 (Figure 6D, Table 1, 30.5%±20.5% increased on R45 as compared to R-1). The ratio of monocytes expressing high level of MHC class II also mildly increased on R45 but decreased nine days after completion (Figure 6D, Table 1). 

### Effects of RR countermeasure

**Figure 7:**
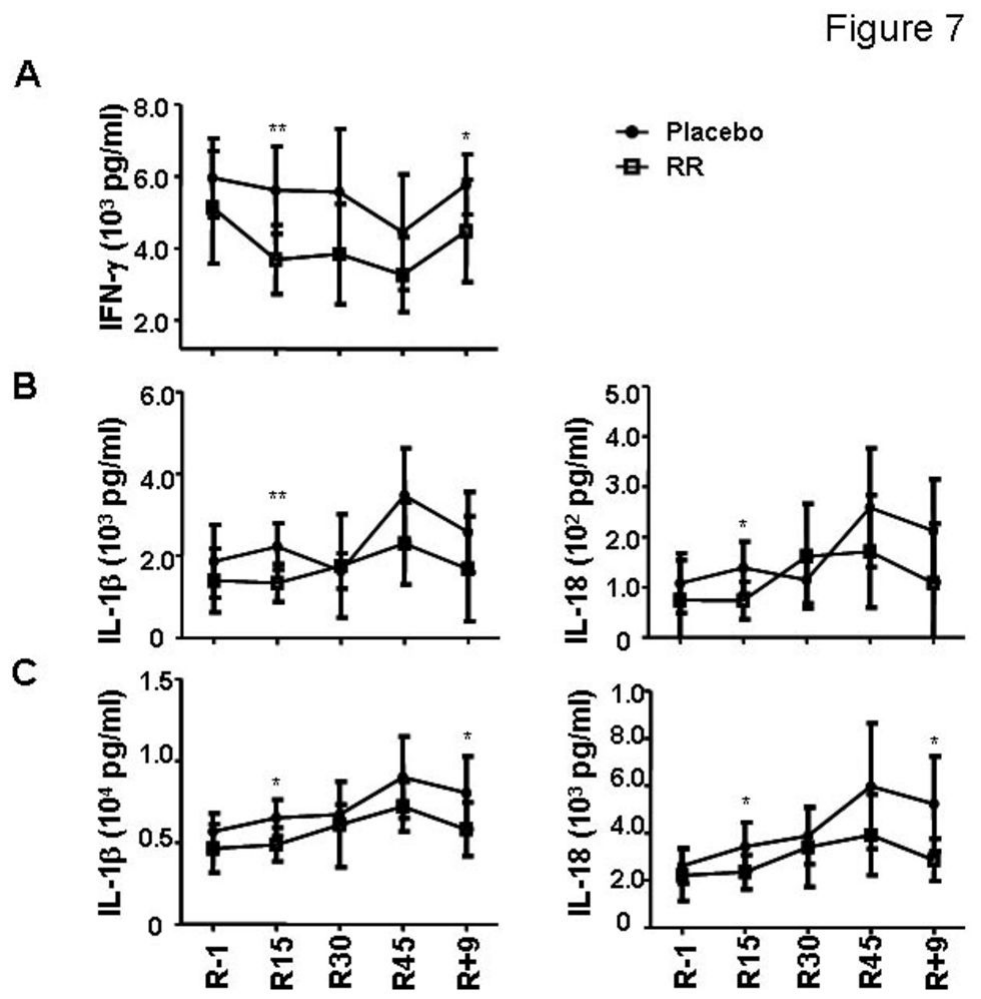
The *Rhodiola rosea* treatment results in further decreased IFN-γ secretion and slowed down the increase of IL-1 family proinflammatory cytokines. (A) The comparison of IFN-γ secretion between the control and RR treated groups. (B) The changes of IL-1β and IL-18 production in response to CpG in RR treated group. (C) The comparison of IL-1β and IL-18 production upon LPS stimulation between the control and RR treated groups. The average percentage of each cell populations at each time point was shown, together with the standard deviation. The statistical significance between the control and RR-treated groups within any single time point was calculated by two-tailed Student *t* test. The following is used to denote *p* values: *p<0.05, **p<0.01, ***p<0.005. The placebo group was shown as filled circle; RR-treated group as open square.

Compared to the placebo group, the adaptogen RR-treated group revealed similar pattern of changes in T, B cell subsets and monocytes. The differentiation of iTreg and the production of antibodies were also similar between the two groups. However, the treatment with RR further decreased the production of IFN-γ (R15) by T cells upon anti-CD3 and anti-CD28 stimulation (Figure 7A, *p* = 0.046 by repeated measures ANOVA, the statistical significance of the data between the control and treated groups was shown in the figure). The secretion of IL-17A was not changed significantly in the group treated with RR. It also significantly slowed down the upregulation of IL-1 and IL-18 on R15 and maintained a lower levels of these two cytokines on R45 and R+9 (Figure 7B and 7C, analysis by repeated measures ANOVA showed that *p* = 0.036 and 0.017 for IL-1β by CpG and LPS, respectively; 0.076 and 0.029 for IL-18 by CpG and LPS, respectively; the statistical significance of the data between the control and treated groups was shown in the figure). 

## Discussion

Spaceflight represents a unique and challenging environment that results in numerous changes in human. The study of the immunoreactivity before, during, and after prolonged and brief flights and even simulated ones are essential for an understanding of integrated responses to microgravity and multiple stresses. In the present study, the simulated weightlessness model (HDBR) revealed similar as well as unique impacts on multiple aspects of human immune system: increased percentages of memory T and B cells, increased percentage of Tregs and monocytes, increased IgE production by B cells, decreased IFN-γ and IL-17 productions by T cells, increased IL-1 family proinflammatory cytokine production by PBMCs. 

Cytokine data from crew members on short- and long-duration spaceflight have shown a significant reduction of IFN-γ and IL-2 [2,52]. In the present 45-day HDBR study, a similar decrease of IFN-γ was found after activation of peripheral blood T cells with anti-CD3 and anti-CD28. In addition, we showed a significant reduction of IL-17A, suggesting a weakened T helper (Th)1 as well as Th17 types of response. Defects in T cell activation or cytokine expression, increased Th2 type cytokine production, increased numbers or suppression function of Treg cells, and/or changes in T cell subset composition in peripheral blood may account for the observed changes. 

It was found that T cells exposed to microgravity have defects in early T cell activation, which is featured by decreased IL-2/IL-2 receptor expression, down-regulated nuclear transcription factor-κB (NF-κB)/Rel and protein kinase A (PKA) activity [24,53,54]. However, our study did not find significant changes in IL-2 and TNF-α production by anti-CD3- and anti-CD28-activated T cells. Furthermore, iTreg differentiation that requires IL-2 signaling was not reduced but increased on R15. This implicates that signaling essential for early T cell activation may not be affected in the present experimental setting. Although we cannot exclude the possibility that any changes in the signaling pathways during microgravity may be recovered during the *in vitro* activation process at 1G, the altered effector molecule expression and iTreg differentiation suggests that microgravity may affect T cell activation far beyond the early phase. 

The microgravity-associated changes in the microenvironment may also have an impact on Th1 and Th17 differentiation. For instance, IL-1β, IL-23, IL-6, and TGF-β all contribute to the generation of Th17 cells and IL-18 promotes IFN-γ production [55-57]. The changes in these cytokines may affect the expression of IL-17 and IFN-γ in T cells. However, serum levels of TGF-β1 were not changed and those of IL-6 were undetectable during the HDBR. Activated immune cells on R45 produced significantly more IL-1β, IL-18, and slightly more TGF-β1, thus neither of them could explain the reduction of IL-17 and IFN-γ secretion upon anti-CD3 and anti-CD28 stimulation. 

The changes of circulating T cell subpopulations may also result in the reduction of effector cytokines. Indeed, the increase of Treg cells was found on R45, the same time point when the levels of IFN-γ and IL-17 production decreased significantly, suggesting that Tregs in the mixed T cell population may be partially involved in suppressing the conventional T cell activation and subsequent cytokine production. The decrease of naïve T cells, IFN-γ- and IL-17-producing memory T cells or γδ T cells may also reduce the production of these two cytokines. However, we found no significant changes in γδ T cell populations and yet an early (R15) decrease of conventional naïve T and increase of memory T cells. Thus, a combination of several microgravity-associated changes, such as an increase of Treg cells and impaired memory T cell function may account for the reduced IFN-γ and IL-17 production at later time points (R45).

The IL-1 family cytokines play a central role in regulating inflammatory responses to both infections and sterile insults such as stress-associated signals. The current study revealed a significant upregulation of IL-1β and IL-18 by LPS- and CpG-activated B cells and myeloid cells at the late phase of HDBR. No significant changes were found in IL-1ra, the antagonist of IL-1 receptor. Similar increase of IL-1 was reported in other HDBR studies [27,28]. The increase of CD14^+^ monocytes in the circulation may account for the upregulation of IL-1 family cytokines. However, the group that received RR treatment exhibited significantly lower IL-1β and IL-18 without changes in monocyte ratios. As an increase of the expression of TLR4 and LPS binding protein (LBP) has been found in astronauts participating in 10-13-day shuttle missions [16], it is possible that the signaling pathways of Toll-like receptors (TLRs) also change during HDBR and subsequently affect the production of proinflammatory cytokines. 

In addition, our data showed a significant increase of concentration of serum cortisol during the HDBR. The activation of neuronal pathways including the hypothalamo-pituitary-adrenal axis (HPA) and the sympathetic nervous system (SNS) may sensitize myeloid cells to produce more IL-1 upon TLR activation [45-47]. Whether it also plays a role in suppressing the production of Th1 and Th17 type cytokines is not clear.

IL-1β is important in the onset and development of a complex inflammatory cascade [56]. The increase of IL-1β was also implicated as one of the factors contributing to the bone decalcification and muscle mass loss during HDBR [27,28]. IL-18 is associated with multiple chronic inflammatory diseases, including atopic eczema, rheumatoid arthritis, systemic lupus erythematosus, and Sjogren’s syndrome [58–60]. Recently, it was also reported that IL-18 can directly induce self-reactive IgM and IgG production and B cells recruitment in the marginal zone of the spleen [61]. Thus, the upregulation of proinflammatory cytokines IL-1 and IL-18 during the long-term HDBR and the slower resolving after the experiment suggest that prolonged microgravity could pose a potential risk in autoimmunity and inflammation. Indeed, mice that had been exposed to the space environment for 91 days showed well correlated increase of IL-1 and inflammatory regions in the testes [62].

The composition of B cell subsets and functions of B cells also altered under microgravity. It has been reported that in urodele amphibian, the transcription of IgY heavy chain was significantly increased, the usage of heavy-chain variable domains of IgM antibodies was altered, and the antibody somatic hypermutation frequency was decreased after a 5-month spaceflight [63-65]. In human, the increase of total IgA and IgM were found after long-term but not short-term missions [8,66,67]. During the present HDBR human study, we further examined the changes of B cell subsets in the peripheral blood. A significant increase of the percentage of resting memory B cells (CD21^+^) but a decrease of activated and tissue like memory B cells were found. It was suggested that the activated and tissue like memory B cells may have enhanced sensitivity to antigens or other stimuli and faster release of effector molecules than naïve or resting memory B cells [68-72]. Individuals with autoimmune diseases or ones infected with pathogen were found to have increased numbers of effector-like B cell subsets. Thus, in addition to decreased antibody somatic hypermutation in amphibian and increased IgA in human under long-term spaceflight conditions, the present HDBR study showed a reduction in “effector” memory B cell population, further support the finding of slow/decreased B cell response to antigens during spaceflights. 

B cells collected from various time points during HDBR were further tested for their antibody responses. Interestingly, only IgE production was increased after CpG stimulation. It is not clear why IgE secretion was upregulated whereas the levels of Th2 type cytokines did not change during HDBR. IL-18 was found to promote the production of IgE [73-75]. Whether the increase of IL-18 upon CpG stimulation plays a role on IgE production awaits further investigation. 

The *Rhodiola rosea* was reported to have an adjuvant effect on immune responses. In the HDBR setting, however, the treatment with RR in vivo further decreased IFN-γ production by T cells and significantly slowed down the upregulation of IL-1 family proinflammatory cytokines by various blood cell types. These immunosuppressive changes brought by RR could be beneficial as slowed IL-1 upregulation may be partially associated with the significant reduction of muscle mass loss found in RR-treated group (Chen, XP et al. manuscript in preparation).

Taken together, the data presented here reveal that a decreased cellular immune response and enhanced sterile inflammatory response occurred during the 45-day HDBR of -6°. The countermeasure of RR treatment reduced the production of proinflammatory cytokines but did not alleviate the cellular immune response. Whether such immune changes result in clinical risks remain to be elucidated. But as most of the significant changes occurred on 45 days after the initiation of HDBR, a higher health risk may be present during long-duration spaceflight.

## Methods

### Ethical issues

The current study was approved by the Ethics Committee of China Astronaut Research and Training Center. Written consent was obtained from the volunteers who had been informed about the risks and the experimental details.

### Subjects

Fifteen male volunteers with 26.63 ± 4.03 (age, presented as means ± SEM), 171.8 ± 3.0 cm (height), and 63.6 ± 6.2 kg (weight) were recruited into this study. All subjects had a junior high education or above. The subjects were healthy and physically fit but were not athletes. Subjects with the following chronic or recent acute illness were precluded: skeleton-muscle diseases, organic and functional diseases of psychiatry, neurology and sleep disorders. None of the subjects had a history of cancer, hepatitis, or any other relevant diseases, including autoimmune disorders. The final selection was based on normal clinical results comprising medical history, physical and psychological examination, complete blood count, blood chemistry analysis and several other tests. Eight and seven of the subjects were randomly selected for the placebo and the RR groups, respectively. The subjects in the RR group take *Rhodiola rosea* 0.50 g per day (prophylactic dose), twice a day from R1 to R7, then 1.0 g per day (therapeutic dose), twice a day from R8 to R45 during HDBR period. The dose of *Rhodiola rosea* was doubled for therapeutic purposes as changes in muscles and bones began to be significant after one week of bed rest. No medication, smoking, alcohol, or caffeinated drinks were allowed during the study. Emergency medical surveillance and service was always available throughout bed rest. 

### Head-down bed rest (HDBR) protocol

The entire experiment started from October 25^th^, 2011 to December 28^th^, 2011. It includes 45 days of HDBR, 10 days of adaptation before HDBR and 10 days of recovery after HDBR. During HDBR, subjects were in resting, flat, head-down position to -6° from the horizontal. Lights were switched off between 2200 and 0600, with normal daylight illumination for the rest of the day. All subjects stayed in an air-conditioned bedroom maintained at 25±0.5°C and relative humidity of 60-70%. All dining, washing, urination and defecation activities were carried out in a bedridden state. Changing position around the body axis was permitted. 

### Blood samples

Sterile heparinized peripheral blood samples were obtained from the fifteen test subjects before (R-1), during (R15, R30, R45) and after (R+9) the HDBR at 6:00 a.m (Figure 1A). Peripheral blood mononuclear cells (PBMCs) were then collected by Ficoll-Hypaque density-gradient centrifugation.

### Reagents

The following mAbs were used for staining: CD14-FITC (M5E2), CD56-PE (B159), CD8-PerCP (SK1) from BD Biosciences (San Jose, CA); CD3-APC (OKT3), CD4-FITC (OKT4), CD45RO-FITC (UCHL1), HLA-DR-PE (L243), CD25-PE (MEM-181) from QuantoBio (Beijing, China); CD4-PerCP-Cy5.5 (OKT4), CD19-PerCP-Cy5.5 (HIB19), CD21-PE (LT21), CD27-APC (O323), CD20-FITC (2H7) from BioLegend (San Diego, CA); and CD27-APC (O323), CD127-FITC (eBioRDR5), CD3-PerCP Cy5.5 (OKT3), γδ TCR-PE (B1.1), CD94-FITC (DX22), Foxp3-APC (PCH101) from eBioscience (San Diego, CA). The following Abs and cytokines were used for cell culture: anti-CD3 (HIT3a), anti-CD28 (CD28.2) from BD Biosciences; rhIL-2, rhTGF-β from R&D Systems; anti-human IgA+IgG+IgM (H+L) from Jackson ImmunoResearch (Suffolk, UK); phosphorothiolated CpG oligodeoxynucleotide type B (CpG-B, ODN 2006) from invivoGen (San Diego, CA); LPS (Eschelichia coli) from Sigma (St Louis, MO). *Rhodiola rosea* containing 0.6% salidroside was purchased from Sihuan Pharmaceuticals (Beijing, China). 

### Phenotypic Analysis

Heparinized blood (0.5 ml) was treated with ACK lysis buffer (0.15 M NH_4_Cl, 10 mM KHCO_3_, 0.1 mM Na_2_EDTA) to remove red blood cells. The cells were then stained with antibodies and examined by a FACSCalibur flow cytometer (BD Biosciences). When lymphocytes were gated from forward and side scatter, the cells with the following phenotypes were analyzed (Figure 5A-C). CD3^+^CD56^-^ was used to analyze conventional T cells. Within this population, naïve T cells were CD4^+^CD45RO^-^ or CD4-CD45RO^-^ (or termed as CD8^+^ naïve T cells); memory T cells were CD4^+^CD45RO^+^ or CD4^-^CD45RO^+^ (or termed as CD8^+^ memory T cells). NKT, CD3^+^CD56^+^ and NK, CD3^-^CD56^+^. γδ TCR and CD3 staining was used to analyze the percentage of γδ T in total T cells. The γδ T cells were further analyzed by the expression of CD27 and CD94. The expression of CD19 was used to analyze B cells. Within CD19^+^ cells, CD21^+^CD27^-^ cells were naïve B cells; CD21^-^CD27^-^ were tissue-like memory B cells; and CD27^+^ were classical memory B cells. The CD27^+^ cells were further divided into CD21^+^CD20^+^ resting memory B, CD21^-^CD20^+^ activated memory B; and CD21^-^CD20^-^ plasmablasts. 

### Serum cytokine and cortisol analysis

Serum samples were sent to LightArray Biotech Research Center (Wuxi, China) for SearchLight Multiplex immuno assay analysis. The cytokines include human TGF-β1, IL-1β, IL-2, IL-4, IL-6, IL-8, IL-10, IFN-γ, TNFα, IL-15, IL-17A, and MCP-1. Serum cortisol was measured using commercially available kits (Alpco Diagnostics, Salem, NH). Samples were batch analyzed to minimize inter-assay variation.

### Cell culture and cytometric Bead Array Analysis

PBMCs were plated at 2 × 10^5^ cells per well of a 96-well flat-bottom plate (Corning, NY) with various combinations of the following reagents: 1 μg/ml anti-CD3 and 1 μg/ml anti-CD28; 10 μg/ml anti-Ig(A+G+M); 2.5 μg/ml CpG-B; or 1 μg/ml LPS. Cells were cultured at 37° in a humidified 5% CO_2_ incubator for 2-3 days before supernatants were removed and kept at -80°C. The cytometric bead array (CBA) was performed according to the manufacturer’s instructions (eBioscience, flowcytomix). IFN-γ, IL-17A, IL-2, IL-10, IL-22, IL-6, IL-4, TNF-α, TGF-β were analyzed by CBA after anti-CD3 and anti-CD28 stimulation; IL-10 and TGF-β after anti-Ig (A+G+M) stimulation; IL-12p70, IL-10, IL-8, IL-6, IL-18, IL-1β, TNF-α, TGF-β after CpG-B stimulation; IL-12p70, MCP-1, IL-10, IL-8, IL-6, IL-18, IL-1β, TNF-α, TGF-β after LPS treatment. Samples were batch analyzed to minimize inter-assay variation

### Immunoglobulin measurement

The supernatant derived from CpG-stimulated PBMCs were sent to Shanghai Yueyan Biological Technology Co. Ltd (Shanghai, China) for the analysis of IgA, IgM, IgE, total IgG, IgG1, IgG2, IgG3, IgG4, and IL-1ra by ELISA.

### iTreg induction

PBMCs were plated at 2 × 10^5^ cells per well of a 96-well flat-bottom plate with 1 μg/ml anti-CD3, 1 μg/ml anti-CD28, 5 ng/ml rhIL-2 and 10 ng/ml rhTGF-β1 for 5 days. The cells were then collected and first stained with CD4 and CD25 antibodies. The intracellular staining of Foxp3 was done according to the manufacturer’s instruction and the cells were examined by a FACSCalibur flow cytometer (Figure 5B).

### Statistical analyses

The data were analyzed by repeated measures ANOVA with time and treatment as two factors for repeated measures and by the post hoc test. The *p* values were shown in the text and Table 1. The statistical significance between any two time points within the control group (Figures 1-4, Figure 6) or between the control and RR-treated groups within a single time point (Figure 7) was further evaluated by two-tailed Student *t* test. The following terminology is used to denote *p* values calculated by Student *t* test: *p<0.05, **p<0.01, ***p<0.005 (Figures 1-4, 6-7). All data are expressed as the mean±standard deviation. Linear regression was used to examine the association between the normalized average cortisol of repeated samples at the same time point and other cytokines. Associations were considered statistically significant when the resulting p value was <0.05 (two-sided). All analyses were performed using SPSS software V.16.0.

## Supporting Information

Figure S1 **Changes of cytokine production by immune cells.** (A) Changes of T cell-derived IL-4 and IL-6 during HDBR. PBMCs were stimulated by anti-CD3 and anti-CD28 antibodies for two days. (B) The production of cytokines by PBMCs upon CpG stimulation. (C) The production of cytokines by PBMCs upon LPS stimulation. PBMCs were stimulated with 1 μg/ml LPS for two days. The supernatants were analyzed by cytometric bead array. Because some of the samples had values out of the linear range of the standard curve, the mean fluorescence intensity (MFI) was used to represent the concentration of the cytokine. The average level of each cytokine at each time point was shown.
(TIF)

Figure S2 **The alteration of T and NK cell percentages during HDBR.** The percentage changes of total T, CD4^+^ and CD8^+^ T cells, and NK cells were shown. The statistical significance between any two time points within the control group was analyzed by two-tailed paired Student *t* test. The following is used to denote *p* values: *p<0.05, **p<0.01, ***p<0.005.
(TIF)
