# Supplementary figures and images for: Changes of Cytokines during a Spaceflight Analog - a 45-Day Head-Down Bed Rest
Source: PLoS One. 2013 Oct 15;8(10):e77401. doi: 10.1371/journal.pone.0077401 (PMC3797033; doi:10.1371/journal.pone.0077401)

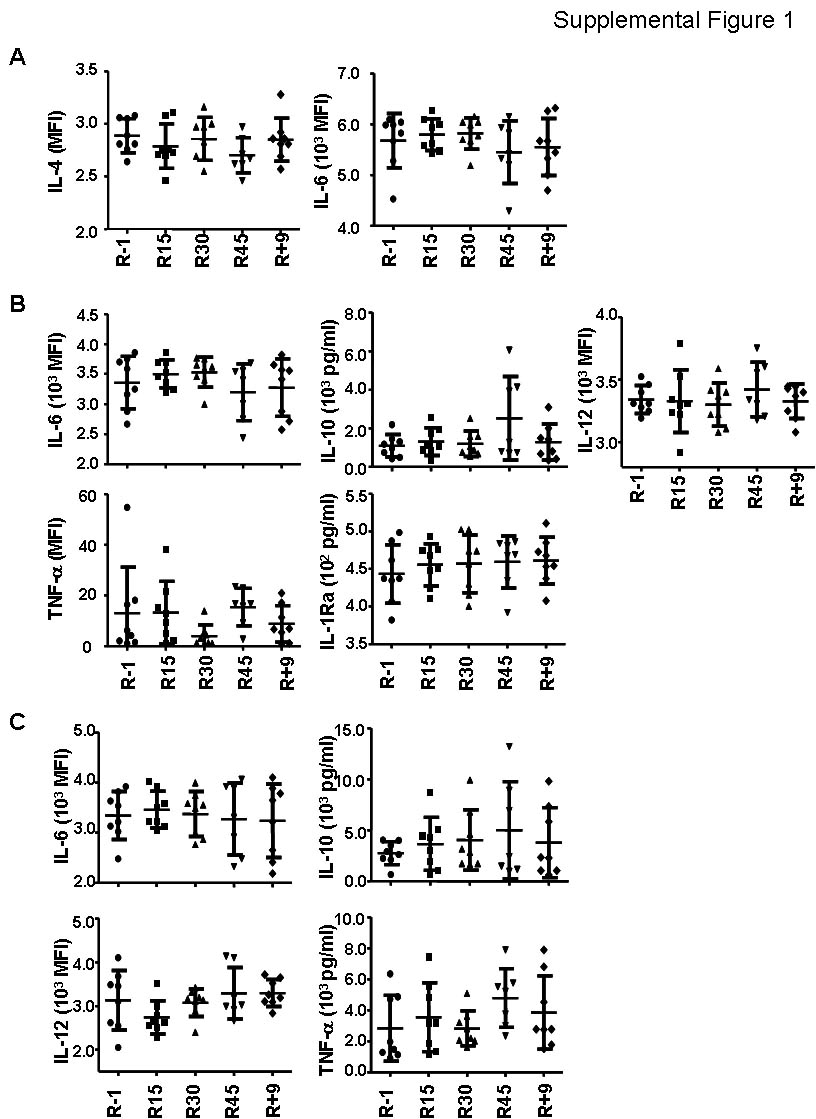

Supplement: Figure S1 — Changes of cytokine production by immune cells. (A) Changes of T cell-derived IL-4 and IL-6 during HDBR. PBMCs were stimulated by anti-CD3 and anti-CD28 antibodies for two days. (B) The production of cytokines by PBMCs upon CpG stimulation. (C) The production of cytokines by PBMCs upon LPS stimulation. PBMCs were stimulated with 1 μg/ml LPS for two days. The supernatants were analyzed by cytometric bead array. Because some of the samples had values out of the linear range of the standard curve, the mean fluorescence intensity (MFI) was used to represent the concentration of the cytokine. The average level of each cytokine at each time point was shown. (TIF) [file pone.0077401.s001.tif]

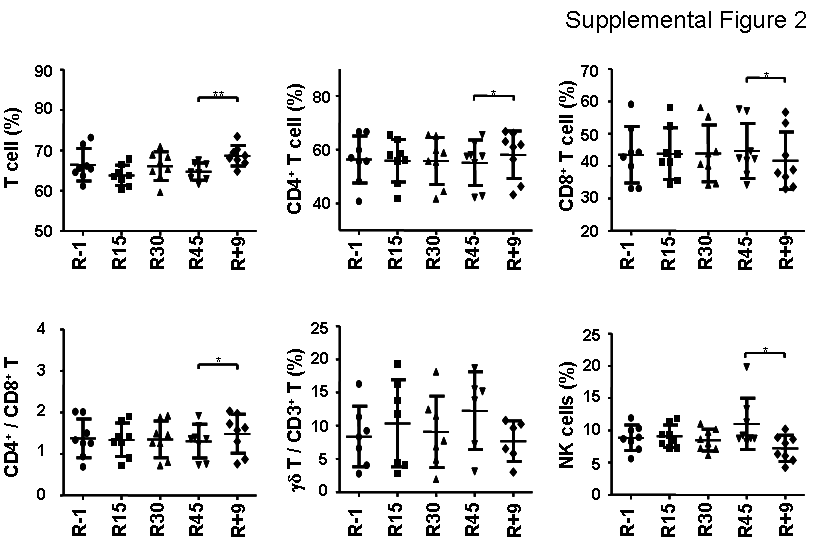

Supplement: Figure S2 — The alteration of T and NK cell percentages during HDBR. The percentage changes of total T, CD4+ and CD8+ T cells, and NK cells were shown. The statistical significance between any two time points within the control group was analyzed by two-tailed paired Student t test. The following is used to denote p values: *p<0.05, **p<0.01, ***p<0.005. (TIF) [file pone.0077401.s002.tif]
